# HIRA: Heart Rate Interval based Rapid Alert score to characterize autonomic dysfunction among patients with sepsis-related acute respiratory failure (ARF)

**DOI:** 10.1088/1361-6579/acf5c7

**Published:** 2023-10-13

**Authors:** Preethi Krishnan, Milad G Rad, Palak Agarwal, Curtis Marshall, Philip Yang, Sivasubramanium V Bhavani, Andre L Holder, Annette Esper, Rishikesan Kamaleswaran

**Affiliations:** 1 Department of Biomedical Engineering, Emory University, Atlanta, GA, Georgia; 2 Department of Biomedical Engineering, Georgia Institute of Technology, Atlanta, GA, Georgia; 3 Department of Biomedical Informatics, Emory University School of Medicine, Atlanta, GA, Georgia; 4 Department of Electrical and Computer Engineering, Georgia Institute of Technology, Atlanta, GA, Georgia; 5 Emory Critical Care Center, Emory University School of Medicine, Atlanta, GA, Georgia; 6 Department of Medicine, Division of Pulmonary, Allergy, Critical Care and Sleep Medicine, Emory University School of Medicine, Atlanta, GA, Georgia

**Keywords:** sepsis, acute respiratory failure, heart rate variability, machine learning

## Abstract

*Objective*. To examine whether heart rate interval based rapid alert (HIRA) score derived from a combination model of heart rate variability (HRV) and modified early warning score (MEWS) is a surrogate for the detection of acute respiratory failure (ARF) in critically ill sepsis patients. *Approach*. Retrospective HRV analysis of sepsis patients admitted to Emory healthcare intensive care unit (ICU) was performed between sepsis-related ARF and sepsis controls without ARF. HRV measures such as time domain, frequency domain, and nonlinear measures were analyzed up to 24 h after patient admission, 1 h before the onset of ARF, and a random event time in the sepsis controls. Statistical significance was computed by the Wilcoxon Rank Sum test. Machine learning algorithms such as eXtreme Gradient Boosting and logistic regression were developed to validate the HIRA score model. The performance of HIRA and early warning score models were evaluated using the area under the receiver operating characteristic (AUROC). *Main Results*. A total of 89 (ICU) patients with sepsis were included in this retrospective cohort study, of whom 31 (34%) developed sepsis-related ARF and 58 (65%) were sepsis controls without ARF. Time-domain HRV for Electrocardiogram (ECG) Beat-to-Beat RR intervals strongly distinguished ARF patients from controls. HRV measures for nonlinear and frequency domains were significantly altered (*p* < 0.05) among ARF compared to controls. The HIRA score AUC: 0.93; 95% confidence interval (CI): 0.88–0.98) showed a higher predictive ability to detect ARF when compared to MEWS (AUC: 0.71; 95% CI: 0.50–0.90). *Significance*. HRV was significantly impaired across patients who developed ARF when compared to controls. The HIRA score uses non-invasively derived HRV and may be used to inform diagnostic and therapeutic decisions regarding the severity of sepsis and earlier identification of the need for mechanical ventilation.

## Introduction

Sepsis is defined as life-threatening organ dysfunction caused by a dysregulated host response to infection (Singer *et al* 2016) that is associated with a high mortality rate and significant short-term and long-term morbidity (Stevenson *et al* 2014, Lelubre and Vincent 2018, Rhee *et al* 2019). One of the major organ dysfunctions that can complicate sepsis is acute respiratory failure (ARF). The causes of ARF in sepsis include the demand for higher minute ventilation to compensate for metabolic acidosis, abnormal compliance, gas exchange due to increased extravascular lung water and pulmonary edema from the capillary leak, and inflammatory lung injury (Rhee *et al* 2019). ARF in critically ill patients with sepsis has been associated with prolonged intensive care unit (ICU) stay, which in turn results in increased morbidity and mortality as well as greater expenditure of healthcare resources (Lai *et al* 2019). As such, there is a clinical need to identify and triage patients with sepsis who are at increased risk of developing ARF.

Although the sequential organ failure assessment (SOFA) and the quick SOFA (qSOFA) scores used in the current Sepsis-3 definition include respiratory components such as the partial pressure arterial oxygen to fraction of inspired oxygen (*P*/*F*) ratio and the respiratory rate, these measures are not standalone indicators of sepsis-related ARF (Singer *et al* 2016). Heart rate variability (HRV) has been studied as a method to risk-stratify and prognosticate patients with sepsis. HRV measures the oscillations of the intervals between consecutive heartbeats and is a non-invasive and indirect evaluation of the autonomic function. Unlike the *P*/*F* ratio and the respiratory rate, HRV is a continuous stream of information that is agnostic to the clinician’s observation or suspicion of a problem. Prior studies have found that HRV can be used to predict not only the risk of developing sepsis, but also several outcomes including the development of septic shock, multiorgan dysfunction, and mortality in patients with sepsis (de Castilho *et al* 2018). However, HRV has not been studied specifically to predict the risk of developing ARF. The modified early warning score (MEWS) is a physiological scoring system that identifies ICU patients at risk of catastrophic deterioration. As a bedside application, a high MEWS score helps in identifying patients who require increased levels of care in the ICU. In response to a high MEWS score, new clinical treatment pathways are created by nurse practitioners and/or critical care physicians (Subbe *et al* 2001). A clinical decision support system based on MEWS has been shown to reduce the length of stay for patients hospitalized with sepsis (Horton *et al* 2020). However, MEWS has lower specificity for predicting adverse outcomes among sepsis patients (Wattanasit and Khwannimit 2021).

Therefore, in this study, we explore the association and implications of HRV and autonomic dysfunction in critically ill sepsis patients. To test our hypothesis HRV was retrospectively compared for the group of sepsis patients that developed ARF requiring the need for invasive mechanical ventilation and a sepsis control group that did not. A newly developed early warning score called heart rate interval-based rapid alert (HIRA), a combination model of HRV and MEWS was compared with MEWS, HRV, and MEWS-lactate scoring systems.

## Study design and methods

**Figure 1.**
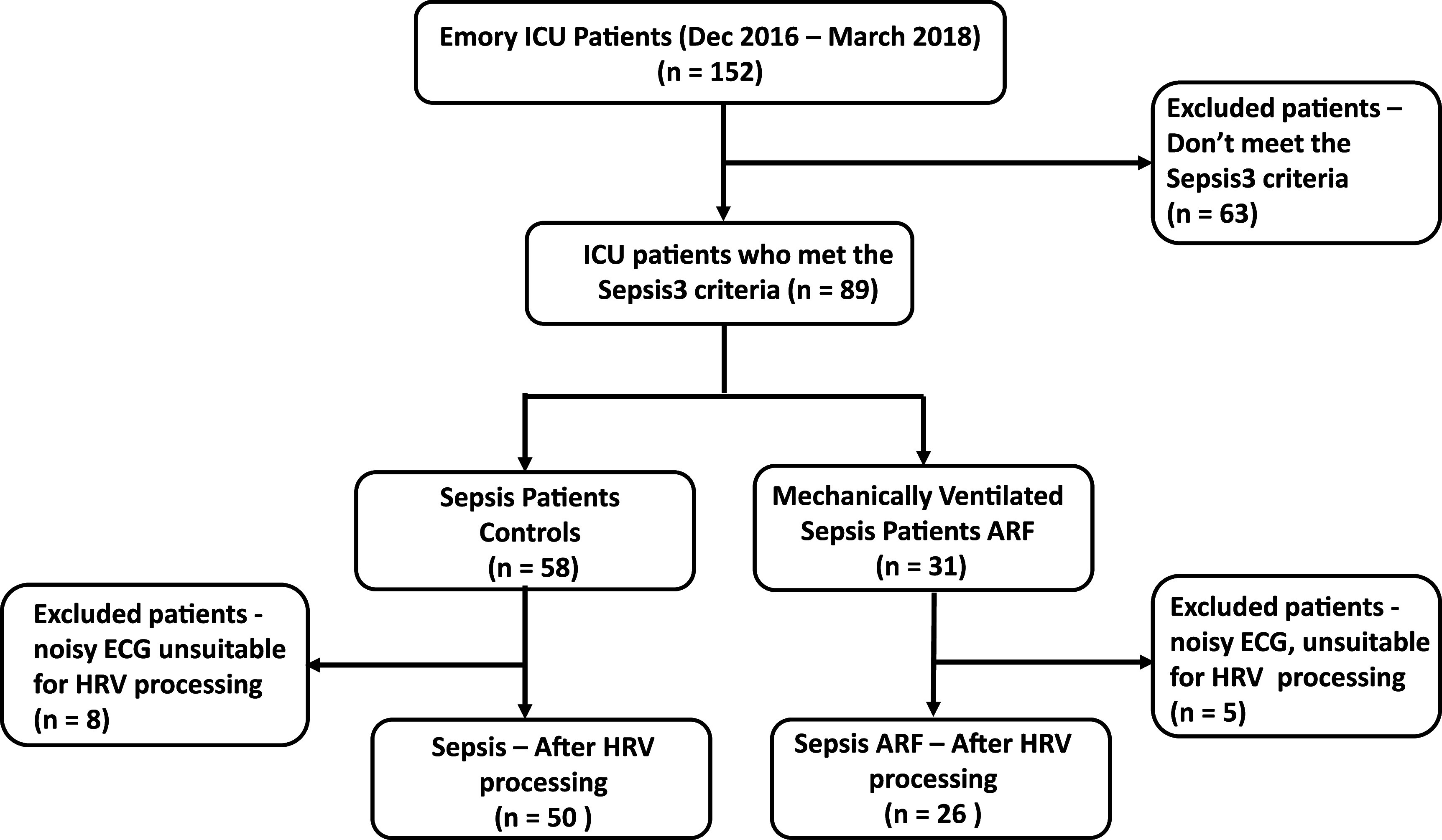
The study population includes all-cause sepsis (controls) and sepsis-related acute respiratory failure (ARF) patients who underwent invasive mechanical ventilation.

This retrospective observational cohort pilot study of sepsis-related ARF patients was approved by Emory University Institutional Review Board, IRB #STUDY00000302. Patients admitted between December 2016 to March 2018 within the Emory Healthcare system from the medical, surgical, neurocritical, transplant, and cardiac ICUs were selected to be part of the study. Physiological data from 152 patient beds were collected from Emory-affiliated hospitals using the general electric (GE) bedside monitors and BedMaster system (Excel Medical Electronics, Jupiter FL, USA). The extracted patient data from bedside monitors consisted of multiple days of the continuous high-frequency ECG waveform. The patient data included in the study was exported from the bedside monitoring archive system and de-identified. The patients were divided into all-cause sepsis patients who met sepsis-3 criteria (1) and developed ARF (requiring invasive mechanical ventilation) and controls were identified as those patients meeting sepsis-3 criteria but did not develop ARF as shown in figure 1. Patients who did not meet the sepsis-3 criteria were excluded from this study.

### Data abstraction

Continuous ECGs were captured from the ICU bedside monitor for the ARF and the control patient cohorts. ECG waveform was sampled at a sampling frequency of 240 Hertz (Hz) and 23 HRV measures were computed using MATLAB^®^ Physionet Cardiovascular Signal Toolbox (Vest *et al* 2018). The HRV measures are described in supplementary table 1. We identified the onset of mechanical ventilation by computing the first presence of a set of ventilator indicator variables (tidal volume, plateau pressure, and PEEP) within the electronic medical record (EMR). The statistical analysis of this data was analyzed using Python scikit-learn software. Statistical significance was computed by the Wilcoxon Rank Sum test and one-way ANOVA.

### Case and control definition

HRV measures calculated over 300 s sliding windows with an overlap of 30 ms were aggregated over two one-hour periods among the sepsis-related ARF cohort, one immediately at ICU admission (as a template for personalized baseline), second one hour 24 h post admission and third one hour before the onset of invasive mechanical ventilation, defined as ARF. To identify an event time among the control cohort, we selected one hour following ICU admission (as a template for personalized baseline) and a second random one-hour period up to 24 h post-ICU admission. This timeframe was selected to adjust for any variation that may arise due to worsening clinical status as opposed to markers that uniquely predict ARF.

### Statistical analysis

The aggregated mean of the event time was adjusted for both controls and ARF using the personalized baseline (admission). The personalized baseline adjusted values were calculated by using the difference between the event value (24 h post admission or one hour before mechanical ventilation) and baseline (admission). The aggregated mean values per patient for HRV features were analyzed using the boxplot. The HRV normalization was performed by dividing event values by the personalized baseline admission values. The resultant values were log_2_ transformed and time indexed to characterize an increase or decrease in the expression of each HRV metric. A heatmap was then generated by applying color-based encoding of the log-transformed data. Color encoding was scaled using the minimum/maximum function to illustrate both increases and decreases in the expression level of the HRV measure relative to the patient baseline. Python seaborn software was used to generate the heat maps and box plot analysis was performed using a method that is a function of the interquartile range. Uniform manifold approximation projection (UMAP) dimensionality reduction technique was evaluated on both controls and ARF using Python UMAP software. Log-transformed aggregated personalized adjusted values (baseline—admission) which are time indexed were used for UMAP visualization.

### Modified early warning score

The MEWS score was calculated using physiological vital signs such as respiratory rate, body temperature, systolic blood pressure, heart rate, and Glasgow Coma Score (GCS) (supplementary table 2). Three MEWS models with thresholds (3, 4, and 5) were calculated using EMR data (supplementary table 3). EMR data for MEWS calculation, including lactate, were imputed using the forward fill method. The MEWS-lactate model is a logistic regression model consisting of MEWS physiological features and blood lactate (Yoo *et al* 2015). The MEWS models were evaluated for controls 24 h after admission and 1 h before mechanical ventilation for the ARF cohort.

### Machine learning models

Logistic regression and eXtreme Gradient Boosting (XGBoost) supervised machine learning methods were used for ARF classification. Synthetic minority oversampling technique (SMOTE) was used to address the imbalanced dataset (Chawla *et al* 2002). Mean aggregated event data without personalized baseline adjustment was used to develop the machine learning model. The leave-one-out cross-validation (LOOCV) K-fold method was used for training and testing the sepsis patient data (Webb *et al* 2011). Grid search methodology was used to perform hyperparameter tuning to determine the optimal values for our model (LaValle *et al* 2004). Bootstrapping was used to estimate the variability of our model’s performance metric, such as the area under the receiver operating characteristic (AUCROC). The HRV model uses XGBoost where HRV statistical measures (supplementary table 1) were used as input features. The HIRA score is an combination model with MEWS and HRV statistical measures. The HIRA model was developed using event data one hour before intubation. The HIRA_ADX (HIRA Admission) model uses event data 24 h post admission. SHapley Additive exPlanation (SHAP) values were used to evaluate the importance of the output resulting from the inclusion of important features (Lundberg *et al* 2017).

## Results

A total of 89 hospitalized ICU patients with sepsis were included in this retrospective cohort study. From this cohort, 31 sepsis patients underwent invasive mechanical ventilation. ECG data one hour after admission, and one hour before invasive mechanical ventilation, were considered for the ARF cohort. The controls consisted of 58 patients who did not require mechanical ventilation. Control (8 patients) and ARF (5 patients) with insufficient ECG data or poor quality have been excluded from this study. The demographic characteristics of ARF patients and controls are described in table 1. The mean age of the ARF and the control group did not differ significantly. There were more males in the ARF cohort, and the number of females was higher in the control cohort. The proportion of African American patients was higher than that of other races among both groups. There was a higher incidence of sepsis among older patients in both groups. Furthermore, sepsis leading to ARF was correlated with higher mortality as shown in table 1.

**Table 1.** Patient demographic characteristics for sepsis-related ARF (Acute Respiratory Failure) and controls (sepsis without ARF).

	Sepsis-related ARF	Sepsis without ARF (Controls)
Patients, *n*	31	58
Valid Data	23 (74%)	58^a^ (100%)^a^
Age, mean [IQR]	55.4 [49.0, 66.0]	59.1[51.0 70.8]
Age Groups, *n* (column %)		
18–40 yrs.	7 (23%)	8 (14%)
41–60 yrs.	11 (35%)	21 (36%)
61–80 yrs.	12 (39%)	25 (43%)
81+ yrs.	1 (3%)	4 (7%)
Male, number (column %)	18 (58%)	27 (47%)
Female, number (column %)	17 (54)	31 (53%)
Race, *n* (column %)		
African American	17 (55%)	34 (59%)
Caucasian	11 (35%)	22 (38%)
Asian	1 (3%)	1 (2%)
Unknown Race	2 (6%)	1 (2%)
In hospital deaths, *n* (column %)	12 (39%)	5 (9%)

^a^ For the 24 h of admit data, 53 (91%) patients had valid data.

**Figure 2.**
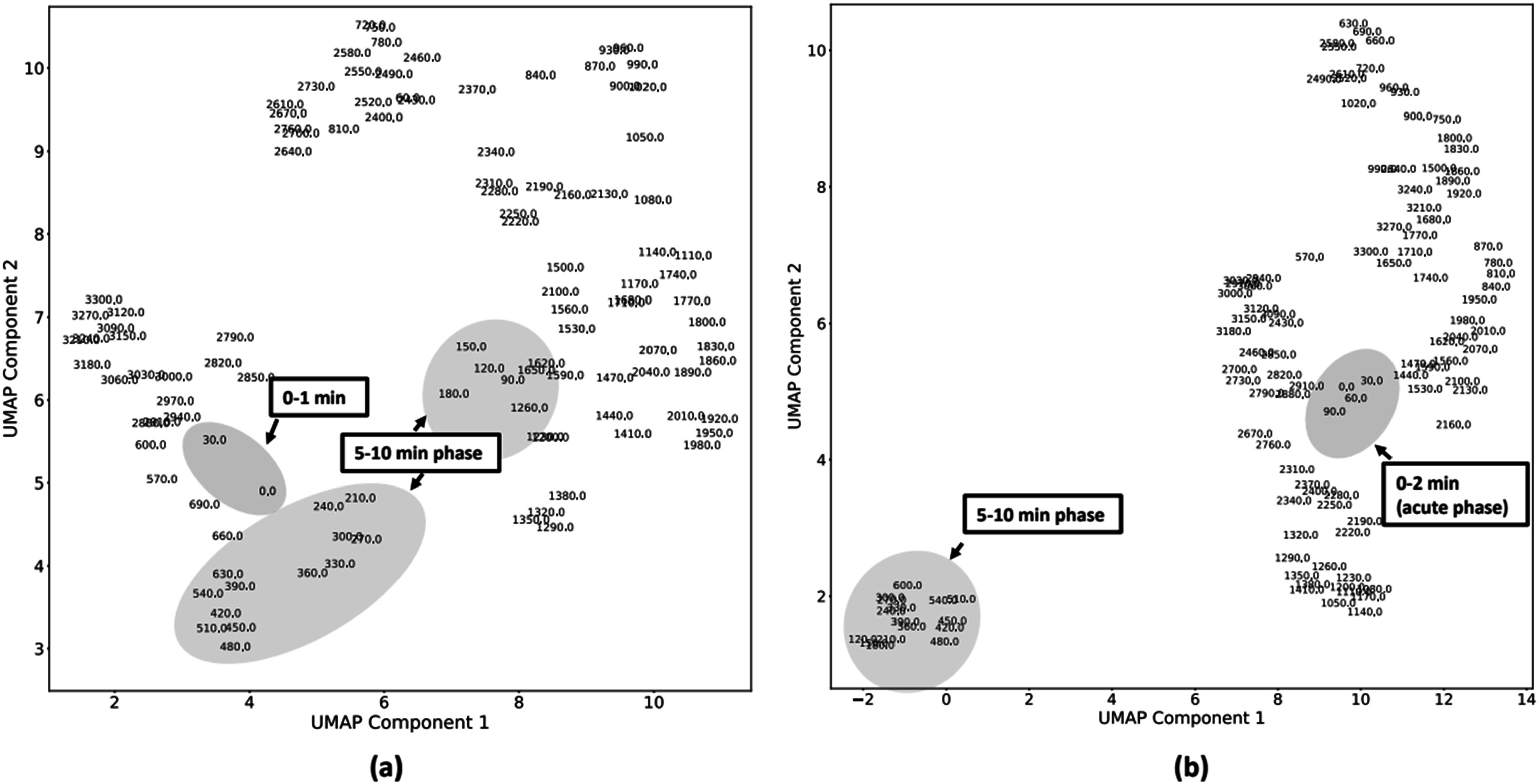
The uniform manifold approximation projection (UMAP) plot shows distinct clustering for controls and the sepsis-related ARF cohort. (A) The controls cohort UMAP plot shows that the temporal regions are organized at random. (B) Sepsis-related ARF cohort UMAP suggests greater dynamics among the HRV features. The clusters within the ARF cohort reveal a grouping of periods between 0 and 2 min before ARF onset (blue highlight region), and a second cluster containing data about an earlier period containing 5–10 min temporal periods.

Table 2 shows the comorbidities and bed unit information for control and ARF cohorts. Both groups showed a higher percentage of renal and cardiac diseases. A higher percentage of control patients were admitted to the medical ICU. For the ARF, a higher number of patients were admitted to the surgical unit. The UMAP plot in figure 2 shows distinct clustering for controls (figure 2(a)) and ARF cohort (figure 2(b)). While two visible clusters are observed in both cohorts, greater separation is seen in the feature space between the ARF cohort, suggesting greater dynamics among the HRV features. Notably, the clusters within the ARF cohort reveal a grouping of periods between 0 and 2 min before ARF onset (blue highlight region, figure 2(b)), and a second cluster containing data about an earlier period containing 5–10 min temporal periods. However, the same temporal regions are organized at random in the control cohort, indicating a possible correlation with physiological deterioration among the ARF cohort. The spatial separation observed in the ARF UMAP may be caused by a combination of the clinical intervention or underlying physiology of sepsis and ARF. We have highlighted the clusters to show the utility of capturing possible physiological dynamics using HRV before/during intubation.

**Table 2.** Patient comorbidities and ICU bed unit for sepsis-related ARF (Acute Respiratory Failure) and controls (sepsis without ARF).

	Sepsis-related ARF (*n* = 31)	Sepsis without ARF (Controls) (*n* = 58)
**Comorbidities**		
Renal failure	20 (64.51%)	42 (72.41%)
Heart failure	17 (54.83%)	29 (50%)
Liver disease	9 (29.03%)	16 (27.5%)
Pneumonia	17 (54.83%)	8 (13.7%)
**ICU Bed Unit**		
Surgical ICU	9 (29.03%)	13 (22.4%)
Cardiac Care Unit (CCU)	8 (29.62%)	18 (31.03%)
Medical ICU	7 (22.58%)	19 (32.75%)
Neuro ICU	7 (22.58%)	8 (13.79%)
**Admit SOFA (mean)**	4.45	2.75

**Figure 3.**
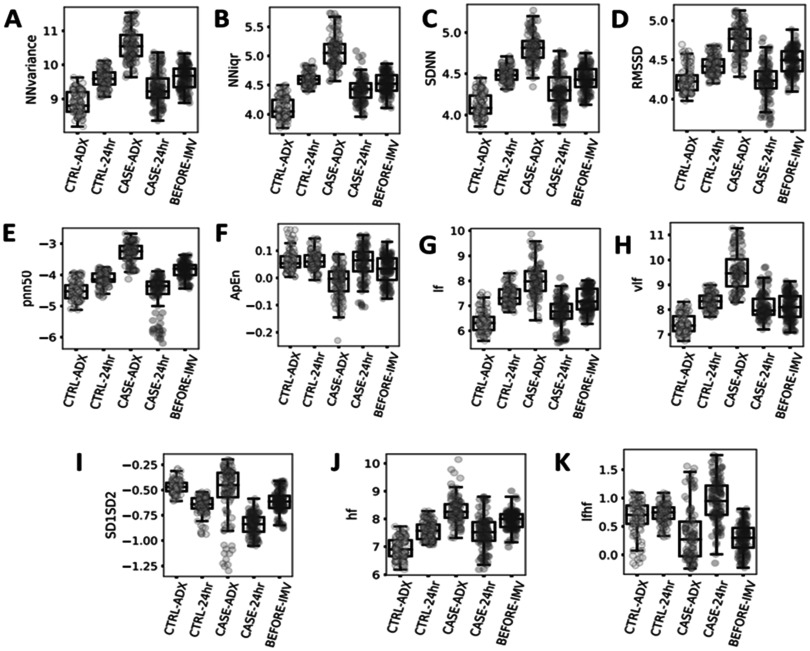
The box-plot panel shows a selected set of time-domain HRV measures that were statistically significant among acute respiratory failure patients in comparison with sepsis patients. The controls cohort includes HRV data 1 h at intensive care unit (ICU) following admission (CTRL-ADX) and 24 h after ICU admission (CTRL-24 h). The ARF cohort includes HRV data for 1 h following ICU admission (CASE-ADX), 24 h after ICU admission (CASE-24 h) and 1 h before invasive mechanical ventilation (BEFORE-IMV). The time-domain HRV measures shown in the figure are as follows: (A) Beat-to-Beat (NN) interval variance (NNvariance). (B) NN interquartile range (NNIqr). (C) NN Standard Deviation (SDNN). (D) Root mean square of successive differences of the NN intervals (RMSSD). (E) Count of NN intervals > 50 milliseconds divided by the total number of all NN intervals (pnn50). (F) Approximate Entropy (ApEn). The frequency domain and nonlinear HRV measures shown in the figure are as follows: (G) low frequency (LF). (H) very low frequency (VLF). (I) SD1/SD2 ratio nonlinear measure (SD1SD2) (J) high frequency (HF). (k) Ratio LF/HF (LFHF).

Figure 3 illustrates a selected set of time-domain HRV box plots that were statistically significant among sepsis patients with and sepsis without ARF. Figure 3(A) shows a statistically significant increase (*P* < 0.05) in beat-to-beat (NN) interval variance among ARF patients one hour before the onset of invasive mechanical ventilation when compared to 24 h post admission. A similar trend is observed in beat-to-beat interquartile range (figure 3(b)), standard deviation (figure 3(c)), root mean square of successive differences (RMSSD) between beats (figure 3(d)), and the percentage of absolute differences in successive NN values > 50 ms (pnn50) as shown in figure 3(e). The beat-to-beat interval metrics such as interval variance and standard deviation show significant lengthening of the RR-interval between ARF and control patients. RMSSD and pNN50 measures relate to the vagal tone. A set of four HRV measures for nonlinear and frequency domain were statistically significant (*P* < 0.05) among sepsis patients with ARF. Frequency measures such as low frequency (LF), very low frequency (VLF), high frequency (HF), and SD1/SD2 ratio nonlinear measure (SD1:SD2) show a statistically significant (*p* < 0.05) decrease for ARF patients (figures 3(g)–(j)) compared to baseline admission. However, approximate entropy (ApEn) shows a statistically significant increase as shown in figure 3(F) respectively.

**Figure 4.**
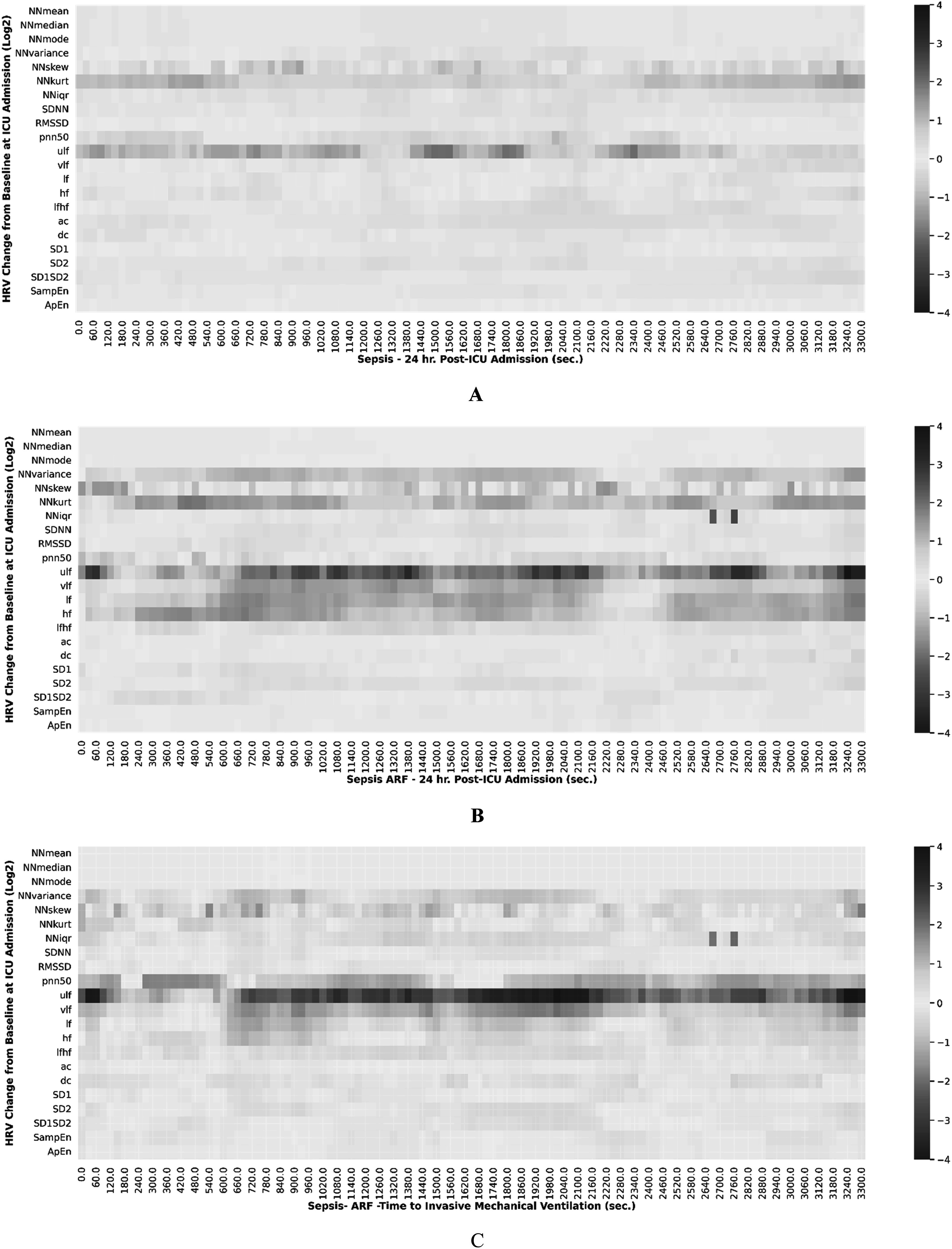
(A) The figure depicts a heatmap of heart rate variability (HRV) statistical measures over time among sepsis patients without ARF at 24 h post ICU admission. NN mean median, mode, and variance were found to be under-expressed. NN kurtosis (kurt) and ultra low frequency (ULF) were over-expressed compared to the baseline at ICU admission. (B) The figure depicts a heatmap of HRV statistical measures over time among sepsis patients with ARF at 24 h post ICU admission. NN variance and frequency measures were over-expressed relative to baseline at ICU admission. (C) The figure depicts a heatmap of HRV statistical measures over time among ARF temporal response in the one hour before the onset of invasive mechanical ventilation (IMV). Frequency measures, NN skew, kurt, and variance show significantly decreased expression.

Figure 4 illustrates a heatmap as a function of HRV dynamics over time among ARF and controls respectively. The heatmap characterizes increased and decreased expression using a personalized benchmark that compares the current magnitude of each HRV measure against the associated metric calculated at 1 h post ICU admission. Figure 4(A) illustrates a similar temporal response among sepsis patients without ARF at 24 h post ICU admission. NN kurtosis and ultra low frequency (ULF) were found to be over-expressed relative to the baseline at ICU admission. Figure 4(B) shows the temporal response among ARF patients 24 h after admission. NN skew is seen to be significantly decreased in a similar timeframe. Additionally, NN variance and frequency measures such as VLF, LF, and HF were over-expressed. Figure 4(C) depicts the temporal expression profile of standard HRV metrics in the one hour before the onset of invasive mechanical ventilation. Several frequency measures and NN skew, kurtosis, and variance show significantly decreased expression 1 h before the onset of ARF.

**Figure 5.**
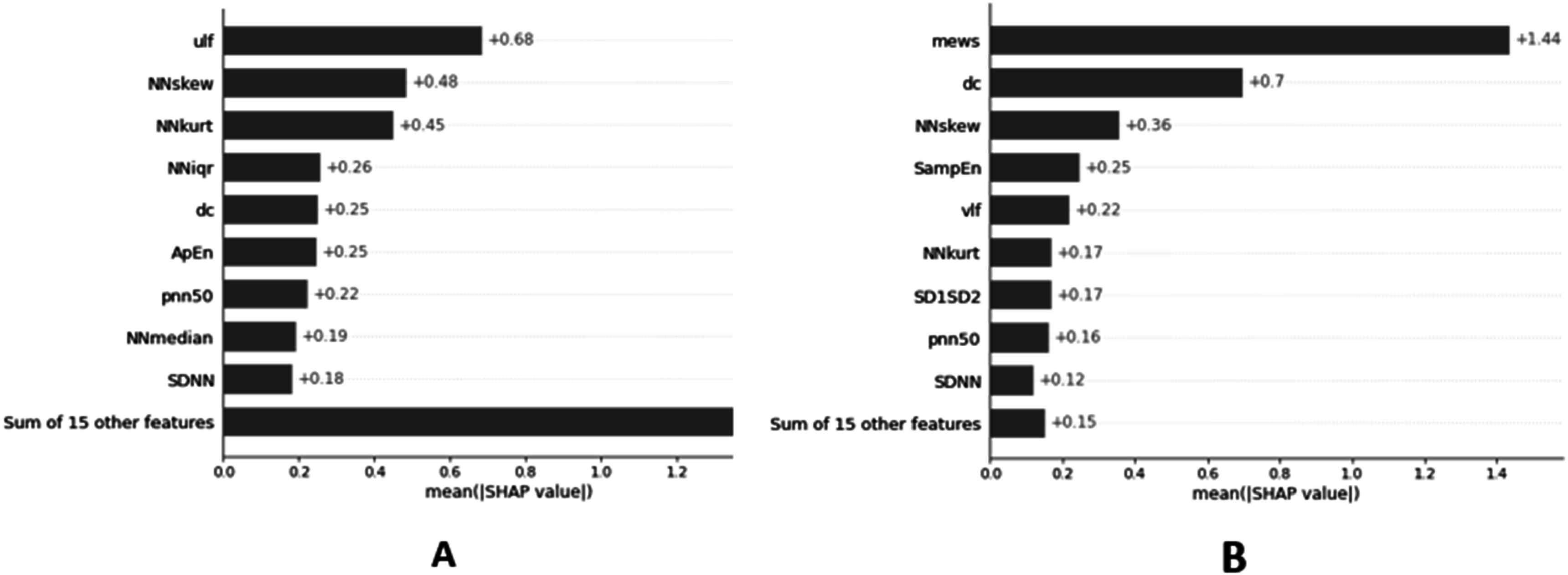
Mean SHAP plot of the top ranked features for ARF prediction (A) HIRA_ADX (24 h post admission) (B) HIRA (1 h before intubation). Abbreviations: ULF, Power in the ultra-low frequency range; NNskew, Skewness of the NN interval; NNkurt, Kurtosis of the NN interval; NNiqr, Interquartile Range of NN intervals; ApEn, Approximate Entropy (a.u.); pnn50, Count of NN intervals > 50 milliseconds divided by the total number of all NN intervals (%); DC, Deceleration capacity (ms); NNmedian, Median of the NN interval (ms); SDNN, Standard deviation of all NN intervals (ms): MEWS, Modified Early Warning Score; SampEn, VLF, Power in the low frequency range; SD1SD2, SD1/SD2 ratio (SD1, SD2 measures of the Poincaré plot), SDNN, Standard deviation of all NN intervals (ms).

**Figure 6.**
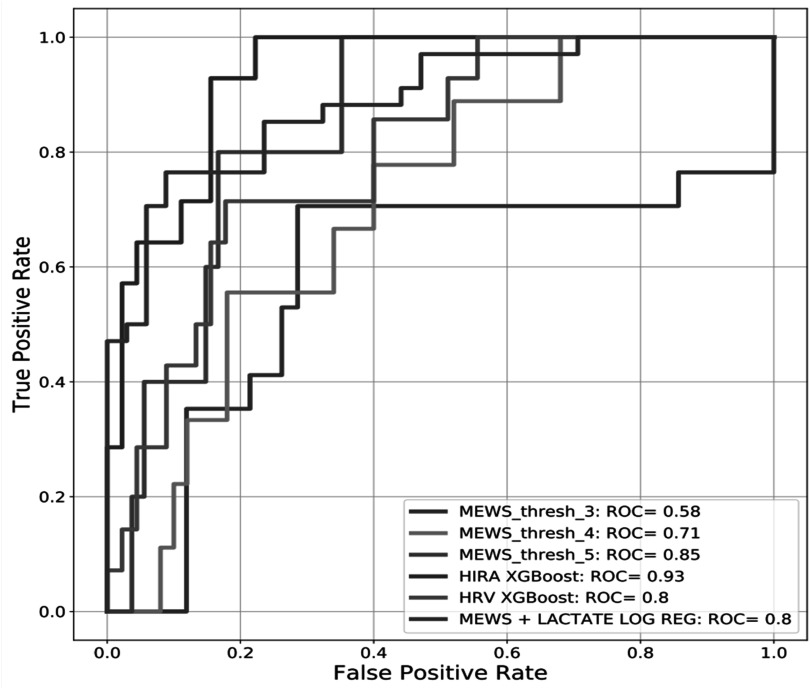
Shows the area under the receiver operating characteristic curve (AUC) curves of the modified early warning score (MEWS) threshold (MEWS_thresh_3, MEWS_thresh_4, MEWS_thresh_5), heart rate interval-based rapid alert (HIRA), heart rate variability (HRV), and MEWS+Lactate ( a combination model of MEWS and lactate). The HIRA XG boost model has yielded the greatest AUC compared to MEWS, MEWS+Lactate, and HRV models.

Table 3 displays the sensitivity, specificity, positive predictive value (PPV), negative predictive value (NPV), and F1 score of the HIRA, HIRA_ADX HRV, MEWS, and MEWS-lactate model. The HIRA model performance metrics (AUC) are higher than the conventional MEWS model which utilized ICU patients’ physiological vital signs. The HIRA_ADX has a higher sensitivity compared to the MEWS models. However, the specificity and PPV are lower. The MEWS-lactate (Sensitivity:0.76, Specificity:0.85) has a higher sensitivity than the HIRA (Sensitivity:0.64, Specificity:0.93) but shows poor specificity indicating the inability to discriminate sepsis patients. Figures 5(A) and (B) shows the important ARF prediction features derived using the relative magnitudes of the mean absolute SHAP values. The HIRA_ADX important features (figure 5(A)) include frequency domain (ULF), time domain (beat-to-beat skew, kurtosis, interquartile range, median, standard deviation, pnn50, approximate entropy, and deceleration capacity. The important features of the HIRA model are also similar including time, and frequency domain HRV features. However, the HIRA model includes additional measures such as nonlinear domain (SD1SD2) and MEWS caused due to physiological changes before intubation or clinical intervention. Figure 6 illustrates the area under the receiver operating characteristic curve (AUC) curves of the HIRA, MEWS (MEWS_threshold_3, MEWS_threshold_4, MEWS_threshold_5) and HRV with AUC, 0.93 (95% confidence interval (CI): 0.88–0.98), 0.58 (95% CI: 0.53–0.78), 0.71 (95% CI: 0.56–0.92, 0.85 (95% CI: 0.50–0.90) and 0.80 (95% CI: 0.64–0.88). HIRA_ADX AUC, 0.64 (supplementary figure 1) was lower than the HIRA developed using the data one hour before intubation. The HIRA model as a combination model of HRV and MEWS outperformed all other scoring systems thereby showing the importance of using non-invasive continuous physiological monitoring in sepsis critical care.

**Table 3.** LOOCV performance metrics of Heart rate Interval-based Rapid Alert (HIRA, combination model of MEWS and Heart rate variability (HRV), 1 h before intubation), HIRA_ADX (24 h post admission), modified early warning score (MEWS) threshold (MEWS_threshold_3, MEWS_threshold_4, MEWS_threshold_5, 1 h before intubation) and MEWS-lactate (a combination model of MEWS and lactate, 1 h before intubation).

Score	Model	Sensitivity	Specificity	Positive predictive value (PPV)	Negative predictive value (NPV)	F1 score
HIRA	XG Boost	0.64	0.93	0.75	0.89	0.69
HIRA_ADX	XG Boost	0.78	0.60	0.37	0.90	0.51
HRV	XG Boost	0.71	0.80	0.52	0.90	0.60
MEWS + Lactate	Logistic Regression	0.76	0.85	0.83	0.78	0.80
MEWS—Threshold - 3	Calculation	0.44	0.91	0.73	0.74	0.55
MEWS—Threshold - 4	Calculation	0.39	0.80	0.47	0.74	0.42
MEWS—Threshold - 5	Calculation	0.30	0.77	0.33	0.74	0.31

## Discussion

The leading causes of ICU admission are sepsis and ARF (Azoulay *et al* 2019). The treatment for ARF frequently involves providing mechanical ventilation; dependent on the balance between oxygen supply, uptake and on adequate ventilation (Wong *et al* 2020). There are currently limited tools available for evaluating patients with sepsis-related ARF. While the MEWS score is normally used as a screening tool to identify sepsis (Evans *et al* 2021), it has low specificity that limits its use in evaluating critically ill sepsis patients (Wattanasit and Khwannimit 2021). In this study, we derived and evaluated supervised machine learning models called HIRA and HIRA_ADX for predicting sepsis-related ARF. By comparing retrospective cohorts of ARF patients and controls, this pilot study demonstrates distinctively expressed subsets of HRV measures that predict the onset of ARF by utilizing the time domain, frequency domain, and nonlinear HRV measures. The results further show statistical separation across several HRV measures between ARF and control patients. The HIRA model achieved higher AUC and predictive value for sepsis-related ARF than HIRA_ADX, MEWS, and MEWS-lactate models and may be used to as a non-invasive method to evaluate ARF patients’ need for invasive mechanical ventilation.

Retrospective HRV data used for the study was one hour and 24 h post-admission for the sepsis control patients. ARF patient retrospective data consisted of 24 h post-admission, HRV data one hour after admission, and 1 h before mechanical ventilation. The interval of 24 h was considered to avoid recording any possible artifacts that might have been generated during ICU admission. Among the significant time-domain HRV measures (supplementary table 1), pNN50, Root mean square of successive difference (RMSSD), standard deviation, interquartile range, variance, and approximate entropy for NN intervals strongly distinguished ARF patients from the control group. However, it should be noted that the significant HRV measures represent overlapping physiological factors and should be considered to be a marker of general indication of autonomic alterations than any individual biology.

pNN50 represents the proportion of pairs of successive NN intervals that differ by more than 50 ms and have been associated with poor autonomic health and an increased risk of mortality. A higher pNN50 value (figure 3(e)) generally indicates greater parasympathetic activity and better autonomic balance. Lower pNN50 values lead to reduced parasympathetic modulation and decreased HRV has been linked to increased cardiovascular risk and poorer outcomes (Kleiger *et al* 2005). SDNN (figure 3(c)) is a commonly used temporal HRV parameter that may be associated with sepsis mortality and reflect all the cyclic components responsible for HRV (de Castilho *et al* 2018). SDNN is also a prognostic indicator of cardiovascular risk in different populations (Chen and Kuo 2007). SDNN regulates the autonomic function in a parasympathetically mediated manner, and the values are influenced by age and associated autonomic nervous system (ANS) function. A higher variance in heart rate indicates a greater degree of variability in the intervals between heart beats (figure 3(a)). The interquartile range (figure 3(b)) is a predictor of mortality (Kleiger *et al* 2005). An increased RMSSD (figure 3(d)) is prevalent in septic shock and could contribute to a potential increase in risk for mortality (Wee *et al* 2020). Approximate entropy is a measure for complex data and identifies changes in time series by assigning a non-negative number to the series. ARF patients at admission showed a lower approximate entropy (figure 3(f)) than the control group, which indicates a decrease in the heart rate modulation dynamics. When considering our HIRA model in the context of existing literature, our results show that there may be valuable pathophysiologic data contained in the HRV data, and that novel machine learning methods such as our HIRA model can utilize this data to provide information about the patients’ clinical trajectory.

LF, HF, LF/HF may be used as a metric to predict malignant ventricular arrhythmias, cardiac death, and arrhythmic death (Arbo *et al* 2020). LF/HF may be an important indicator of ANS activity in sepsis but further studies are needed to show molecular markers from biospecimens to understand pathophysiologic implications of our data. Future studies with HRV data for 24 h or more will be required to understand the association between frequency domain features and autonomic dysfunction among critically ill sepsis patients. SD1 and SD2 are nonlinear measures which is defined as the standard deviation measuring the dispersion of points in the plot perpendicular to the line of identity. SD1SD2 is derived from the Poincaré Plot and is a measure of autonomic balance. An increased SD1SD2 as shown in figure 3(i) may indicate broad and complex dynamics between the sympathetic and parasympathetic arms. The ratio of SD1/SD2 measures unpredictability and is used to measure autonomic balance when there is sympathetic activation (Mazzuco *et al* 2015, Shaffer and Ginsberg 2017).

In our study, machine learning models were developed for two event times: 1 h before mechanical ventilation (HIRA) and 24 h post-admission (HIRA_ADX). The HIRA model (sensitivity-0.64, AUC −0.93) performed significantly better in predicting ARF in comparison to MEWS (sensitivity-0.44, AUC-0.58). Additionally, HIRA_ADX (sensitivity—0.78. AUC-0.64) also showed higher sensitivity and AUC (supplementary figure 1) however, further patient HRV trajectory studies are required to optimize the model performance. A combined model of blood lactate and MEWS has shown its usefulness in predicting ICU transfer in critically ill sepsis patients (Yoo *et al* 2015). Conventionally blood lactate is measured infrequently using invasive techniques in an ICU setting, usually coinciding with the suspicion of infection. HRV, on the other hand, can be computed continuously using the non-invasive ECG physiological signal obtained from the ICU bedside monitor, and thus has the advantage of capturing useful clinical data continuously and noninvasively. Moreover, its superior performance in identifying ARF compared to MEWS and MEWS-lactate in our pilot study demonstrates its future potential as an important clinical decision support tool. The feature importance of SHAP plots helps in interpreting our HIRA model and provides clarity regarding the important clinical features for sepsis-related ARF prediction. The feature visualization techniques allow clinicians to trust the model predictions and understand its mechanisms. HIRA score with its improved predictive performance and ease of use can provide additional patient information to clinicians. As a combination model incorporating HRV and vital physiological signals, the HIRA score has the potential to inform diagnostic and therapeutic decisions regarding the severity of sepsis and ARF.

### Limitations

A smaller group of sepsis patients from a single urban academic center was considered for this study. Factors such as body position, respiratory rate, and mechanical ventilation weaning can influence HRV measures. The HIRA model was not evaluated on an external validation dataset. Another limitation is that approximately 40% of the ARF group required mechanical ventilation for less than 24 h, and it is possible that these patients were ventilated for reasons other than true ARF. While the majority of ARF patients were started on mechanical ventilation up to 10 d after admission, there were some patients who were intubated many days into the admission and could have represented different pathophysiology than ARF patients who were intubated earlier in their hospitalization. ARF recovery outcomes influenced by the above factors were not part of the HRV analyses. Pharmacological interventions and the influence of comorbidities will be evaluated in our future work.

## Data Availability

The data cannot be made publicly available upon publication due to legal restrictions preventing unrestricted public distribution. The data that support the findings of this study are available upon reasonable request from the authors.

## Grant

RK was funded by the national institute for general medical sciences of the National Institutes of Health (NIH) under R01GM139967, and National Center for Advancing Translational Sciences (NCATS) under UL1TR002378 and the Surgical Critical Care Initiative (SC2i), Department of Defense Health Program Joint Program Committee 6/Combat Casualty Care (USUHS HT9404-13-1-0032 and HU0001-15-2-0001). PY is supported by National Heart, Lung, and Blood Institute (NHLBI 5T32HL116271-08). SVB is supported by the National Institute for General Medical Sciences of the National Institutes of Health ( K23GM144867). AH is supported by the National Institute of General Medical Sciences of the National Institutes of Health (K23GM37182). The content is solely the responsibility of the authors and does not necessarily represent the official views of the National Institutes of Health.

## Author contributions

PK—Writing—Original Draft, Methodology, Visualization, Conceptualization. MR-Original Draft, Methodology, Data Curation. PA - Original Draft, Methodology, Data Curation. CM—Writing—Original Draft, Methodology, Data Curation. PY—Writing—Original Draft, Methodology, Supervision, SVB—Methodology, Supervision, Writing—Review and Editing. AH—Methodology, Supervision, Writing—Review and Editing. AE—Methodology, Supervision, Writing—Review and Editing. RK—Conceptualization, Methodology, Supervision, Project administration, Writing—Original Draft, Visualization, Guarantor.
